# A synthetic system links FeFe-hydrogenases to essential *E. coli *sulfur metabolism

**DOI:** 10.1186/1754-1611-5-7

**Published:** 2011-05-26

**Authors:** Buz Barstow, Christina M Agapakis, Patrick M Boyle, Gerald Grandl, Pamela A Silver, Edwin H Wintermute

**Affiliations:** 1 Department of Systems Biology, Harvard Medical School, Boston MA 02115, USA; 2 Wyss Institute, Harvard University, Boston MA 02115, USA

## Abstract

**Background:**

FeFe-hydrogenases are the most active class of H_2_-producing enzymes known in nature and may have important applications in clean H_2 _energy production. Many potential uses are currently complicated by a crucial weakness: the active sites of all known FeFe-hydrogenases are irreversibly inactivated by O_2_.

**Results:**

We have developed a synthetic metabolic pathway in *E. coli *that links FeFe-hydrogenase activity to the production of the essential amino acid cysteine. Our design includes a complementary host strain whose endogenous redox pool is insulated from the synthetic metabolic pathway. Host viability on a selective medium requires hydrogenase expression, and moderate O_2 _levels eliminate growth. This pathway forms the basis for a genetic selection for O_2 _tolerance. Genetically selected hydrogenases did not show improved stability in O_2 _and in many cases had lost H_2 _production activity. The isolated mutations cluster significantly on charged surface residues, suggesting the evolution of binding surfaces that may accelerate hydrogenase electron transfer.

**Conclusions:**

Rational design can optimize a fully heterologous three-component pathway to provide an essential metabolic flux while remaining insulated from the endogenous redox pool. We have developed a number of convenient *in vivo *assays to aid in the engineering of synthetic H_2 _metabolism. Our results also indicate a H_2_-independent redox activity in three different FeFe-hydrogenases, with implications for the future directed evolution of H_2_-activating catalysts.

## Background

Nature provides a catalyst for H_2 _production: hydrogenase metalloenzymes[1]. Hydrogenase active sites are composed only of widely available metals, yet can reduce protons to H_2 _more rapidly than platinum catalysts[2]. They have therefore been proposed as a substitute for precious metal electrodes for the low-cost, high-volume interconversion of electricity and H_2_[2,3]. Enzymatic catalysts are particularly well-suited for synthetic integration with specifically biological energy sources. For example, coupling of hydrogenase enzymes with the photosynthetic machinery enables the direct production of H_2 _from sunlight, as has been demonstrated both *in vitro*[4] and *in vivo*[5,6].

Several classes of hydrogenases are found in nature[1], distinguished primarily by the metal content of their active site. These enzymes vary widely with respect to their activity level, maturation requirements, redox partners and O_2 _tolerance, all of which affect their potential biotechnological utility.

Here we employ iron-iron (FeFe) hydrogenases of the monomeric, cytosolic class found widely in nature and common among the Clostridia[7]. These are the biochemically best-characterized hydrogenases, featuring a well-defined set of maturation factors that are sufficient for their heterologous expression in engineered organisms[8,9]. Members of this class exhibit among the highest activities of known hydrogenases, with catalytic rates exceeding the 1000 nmol H_2 _mg^-1 ^min^-1 ^estimated to be required for efficient *in vivo *photosynthetic H_2 _production[10]. However, biotechnological applications of FeFe-hydrogenases are impeded by the rapid and irreversible inactivation of the active site by O_2_[11].

In contrast, several naturally occurring hydrogenases of the nickel-iron (NiFe) class[12-15] and the nickel-iron-selenium (NiFeSe) class[16] are known to be O_2_-tolerant. However, the H_2 _production rates of NiFe-hydrogenases are 10 to 1000 times slower than FeFe-hydrogenases[1,17,18]. The maturation requirements for NiFe-hydrogenases are also relatively complex, with as many as 13 genes required for heterologous[1,17,18] reconstitution in some cases[19], limiting their facility of heterologous expression.

There is currently no known hydrogenase of any class that produces H_2 _at rates comparable to the FeFe-hydrogenases and is tolerant of O_2_. However, both the activities and O_2 _tolerance of naturally occurring FeFe-hydrogenases span wide ranges and are uncorrelated[20]. O_2 _sensitivity may even be adaptive in hydrogenase evolution, acting as a form of regulation[21,22]. If the natural selective pressure for O_2 _tolerance is weak, it should be possible to engineer a hydrogenase with both high activity and improved O_2 _tolerance.

Recent results suggest structural modifications that may alter hydrogenase O_2 _tolerance. Widening a gas channel, through which H_2 _is believed to approach the active site, resulted in the loss of O_2 _tolerance in a *Ralstonia eutropha *NiFe-hydrogenase[23]. Similarly, the mutation of gas channel residues in a *Desulfovibrio fructosovorans *NiFe-hydrogenase increases O_2 _tolerance yet only moderately reduces catalytic activity[24]. Although understanding of the mechanism of O_2_-inactivation of hydrogenases has greatly improved [25,26] it has not yet been possible to rationally design mutations to improve O_2 _tolerance without impairing activity.

Directed evolution permits the optimization of function without detailed knowledge of enzyme mechanism[27,28]. This technique entails either screening or genetically selecting for desirable mutants from suitably large mutation libraries. *In vitro *screens for hydrogenase activity are capable of assaying 10^3 ^- 10^4 ^hydrogenase mutants per day [29]. Yet double or triple combinatorial point mutation libraries for a gene the size of the hydrogenase approach 10^7 ^- 10^10 ^unique sequences, and can not be comprehensively screened with existing methods. Genetic selection is the most powerful available tool for the rapid and economical parsing of very large mutation libraries. This technique requires a connection between a desired enzyme property and the evolutionary fitness of a strain expressing the enzyme.

We have engineered an artificial pathway in *E. coli *connecting hydrogenase function to the production of sulfide, an essential precursor of the amino acids cysteine and methionine. We address two design objectives that maximize the utility of this system for directed evolution. First, we minimize pathway activity independent of the hydrogenase, insulating redox interactions with native metabolism and reducing the potential for selection false positives. Second, we maximize hydrogenase-dependent activity of the pathway, creating a robust link to host fitness by ensuring binding compatibility between synthetic pathway components. Because genetic selection can be performed only *in vivo*, we have optimized the biochemistry of our pathway *in vivo*. This pathway forms the basis of a genetic selection for O_2_-tolerant hydrogenases.

## Methods

### Cloning and gene synthesis

All cloning was performed in *E. coli *DH5α using standard BioBrick assembly techniques[30]. Final constructs were assembled in commercial Duet vectors (Novagen) with multiple cloning sites modified to accept BioBrick parts. The plasmids used in these experiments are listed in table 1. Complete vector sequences are provided in additional file 1.

**Table 1:** Plasmids used in this study

Name	Constructs		Backbone	Resistance	Source
**Hydrogenase activity *in vivo*^A^**					

pET.mp1	*ca*HydE	*ca*HydA	pETDuet-1	Ampicillin	Matthew Posewitz[31]

pCDF.mp2	*ca*HydF	*ca*HydG	pCDFDuet-1	Spectinomycin	Matthew Posewitz[31]

pACYC.ew3	*da*PFOR		pACYCDuet-1	Chloramphenicol	This work

pACYC.ew4	*da*PFOR	*so*FD	pACYCDuet-1	Chloramphenicol	This work

pACYC.ew5	*da*PFOR	*zm*FD	pACYCDuet-1	Chloramphenicol	This work

pACYC.ew6	*da*PFOR	*cr*FD	pACYCDuet-1	Chloramphenicol	This work

pACYC.ew7	*da*PFOR	*ca*FD	pACYCDuet-1	Chloramphenicol	This work

pACYC.ew8	*so*FD		pACYCDuet-1	Chloramphenicol	This work

pACYC.ew9	*zm*FD		pACYCDuet-1	Chloramphenicol	This work

pACYC.ew10	*cr*FD		pACYCDuet-1	Chloramphenicol	This work

pACYC.ew11	*ca*FD		pACYCDuet-1	Chloramphenicol	This work

**FNR-supported growth^B^**					

pCDF.ew12	*zm*FNR		pCDFDuet-1	Spectinomycin	This work

pACYC.ew13	*so*FD	*zm*SIR	pACYCDuet-1	Chloramphenicol	This work

pACYC.ew14	*zm*FD	*zm*SIR	pACYCDuet-1	Chloramphenicol	This work

pACYC.ew15	*cr*FD	*zm*SIR	pACYCDuet-1	Chloramphenicol	This work

pACYC.ew16	*ca*FD	*zm*SIR	pACYCDuet-1	Chloramphenicol	This work

pACYC.ew17	*zm*SIR		pACYCDuet-1	Chloramphenicol	This work

**Hydrogenase-supported growth and selection^C^**					

pACYC.ew18	*cr*HydEF	*cr*HydG	pACYCDuet-1	Chloramphenicol	This work

pET.ew19	*so*FD	*zm*SIR	pETDuet-1	Ampicillin	This work

pCDF.ew20	*cr*HydA		pCDFDuet-1	Spectinomycin	This work

pCDF.ew21	*ca*HydA		pCDFDuet-1	Spectinomycin	This work

pCDF.ew22	*cs*HydA		pCDFDuet-1	Spectinomycin	This work

Duet vector backbones (Novagen) were used for all protein expression. Complete vector sequences are provided in additional file 1. A) Plasmids mp1-ew11 were used to generate PFOR-driven hydrogenase activity data for Figure 4. B) Plasmids ew12-ew17 were used to assess knockout strain insulation for Figure 2. They were also used to measure ferredoxin performance in Figure 4. C) Plasmids ew18-ew22 facilitated the hydrogenase O_2 _tolerance measurements in Figure 3. They were also used in the hydrogenase-supported growth curves in Figure 5 and for the genetic selection.

The hydrogenase genes *hydA1 *from *Clostridium acetobutylicum *ATCC 824 and *hydA *from *Clostridium saccharobutylicum *P262 were cloned from plasmids received from Matthew Posewitz (National Renewable Energy Laboratory, Golden, CO, USA). Dr. Posewitz also provided plasmids bearing the *C. acetobutylicum *maturation factors *hydE*, *hydF*, and *hydG*[31]. The hydrogenase maturation factors *hydEF *and *hydG *from *Chlamydomonas reinhardtii *were commercially synthesized by Codon Devices. The *por *gene encoding pyruvate-ferredoxin oxidoreductase from *Desulfovibrio africanus *was cloned from plasmid pLP1[32] provided by Laetitia Pieulle (Centre National de la Recherche Scientifique, Marseille, France). *Clostridium acetobutylicum *ferredoxin CAC0303[33] was cloned from genomic DNA (ATCC 824). *Spinacia oleracea *ferredoxin (GenBank AAA34028), *Zea mays *ferredoxin (GenBank ACA34367) and *Zea mays *sulfite reductase (GenBank BAA23641) were cloned from total RNA. The ferredoxin-NADPH reductase (FNR) gene from *Zea mays *(GenBank AAB40034) was synthesized by Codon Devices. Chloroplast transit peptides were omitted from all plant-derived constructs. The codon usage of synthetic genes was optimized by the manufacturer for heterologous expression.

### Gene expression

All synthetic pathways were expressed in strains derived from *E. coli *BL21(DE3), obtained from Agilent Technologies. Plasmids were maintained through selective antibiotics: ampicillin, 50 μg/mL; spectinomycin, 25 μg/mL; kanamycin, 25 μg/mL; chloramphenicol, 12.5 μg/mL. Protein expression from the Duet vectors was T7-promoter driven and induced with IPTG at a concentration of 1 mM.

### Gene deletions

Sequential gene deletions were constructed by P1 phage transduction from the Keio collection[34]. Serial deletions were enabled by removing the transduced kanamycin resistance marker though homologous recombination at flanking FRT sites. Transient expression of the Flp recombinase was facilitated by the 705-Flp plasmid, which exhibits temperature-sensitive recombinase expression and a temperature-sensitive replication origin[35]. Kanamycin marker integration and subsequent removal was confirmed for all loci by PCR.

### Selective and induction media

Selective media was a standard M9 formulation, supplemented with additional glucose, sulfate, ferric iron and a rich mix of supplements less cysteine and methionine. Induction media for hydrogenase expression was LB with added glucose, ferric iron, phosphate buffer and Baker's antifoam reagent. Exact media recipes are provided in additional file 2.

### Anaerobic technique and custom atmospheres

Anaerobic liquid culture was performed in 40 mL serum vials sparged with N_2 _and sealed with SubaSeal^® ^rubber septa (Sigma-Aldrich). To maintain anaerobiosis during handling, samples were drawn and reagents added by piercing the septa with non-coring syringe needles.

Agar plates were incubated under defined gas mixtures within sealed Vacu-Quick jars (Almore International). The ambient atmosphere was removed by several cycles of evacuation and replacement with pure N_2 _before supplying a custom atmosphere. Aluminosilicate desiccant packets were added to prevent moisture accumulation within the jars.

### Growth assays

Cells were grown to saturation in induction media and washed 3× with phosphate-buffered saline (PBS). Cells were resuspended in selective media at an initial OD_600 _of 0.01. Final ODs were measured after 18 hours of growth at 37°C. Anaerobic conditions, when appropriate, were introduced as described above.

### *In situ *hydrogenase activity assays

Cells were grown to saturation in anaerobic induction media. Samples were drawn to determine cell density by OD. Fresh serum vials containing 25 mL of induction LB were anaerobically inoculated with 10^8 ^cells (≈ 5 mL). Following incubation at 37°C for 2 hours, H_2 _production was stopped by the addition 2 mL methanol. Accumulated headspace H_2 _was measured by gas chromatography (Shimadzu GC-14A).

### *In vitro *hydrogenase activity assays

Hydrogenase activities were measured with a biochemical methyl viologen assay adapted from King *et al*.[9]. Hydrogenase-expressing *E. coli *were grown to saturation in 20 mL of induction media under anaerobic conditions. Samples were drawn to determine cell density by OD. Cells were lysed with 1 mL of lysis buffer consisting of 20 mL B-PER II protein extraction reagent (Thermo Scientific), 500 μL Baker's antifoam B, 100 units DNAse I and 50 mg dithionite. Antifoam and DNAse I were added to prevent foaming of the lysate. Dithionite served to scavenge dissolved O_2 _in the buffer. Lysis continued for 15 minutes under continuous N_2 _sparging. Lysed cultures were sealed and injected with 1 mL of methyl viologen assay buffer consisting of 20 mL 1 M Tris at pH 8, 300 mg methyl viologen and 3 g dithionite. Following 2 hours of incubation at 37°C, H_2 _production was stopped with 2 mL of methanol. Accumulated headspace H_2 _was measured by gas chromatography.

### Sulfide production assays

Sulfide production was measured spectrophotometrically by the methylene blue method[36]. Bacteria were grown to saturation in selective media with IPTG and appropriate antibiotics, supplemented with 100 mg/L cysteine to allow growth of all strains. Cultures were diluted 1:50 to 25 ml in 40 ml sealed serum vials. Vials were flushed with either pure N_2 _or 40% H_2 _in N_2 _and grown for 6 hours at 37°C. Cuvettes were prepared with 2.5 mL assay solution containing N,N-Dimethyl-1,4-phenylenediammonium dichloride (200 μM), FeCl (600 μM) and HCl (60 mM). 25 μL of filtered media was added to assay cuvettes and allowed to react for 1 hour. Absorbance was measured at 660 nm and compared to a standard curve to calculate sulfide concentrations. Statistics were inferred from three biological replicates.

### Hydrogenase O_2 _tolerance assays

Hydrogenase half-lives in O_2 _were measured using an adaptation of the *in vitro *activity assay above. Following anaerobic lysis, a customized sparging apparatus was used to bubble O_2 _through the cultures at a constant partial pressure of 0.1 atm for defined time points between 5 and 25 minutes. Cultures were then flushed for 5 minutes with pure nitrogen before being sealed and assayed with methyl viologen assay buffer, as above. Measurements at each time point were taken for 3 biological replicates.

### Colony growth assays under custom atmospheres

Cells were grown to saturation in induction media. Residual nutrients were removed by 3× washing with PBS. Cell densities were measured with a hemocytometer and diluted to a final concentration of 1 cell/μL. Fifty microliters (≈ 50 cells) were dispensed onto selective plates and transferred to Vacu-Quick jars. The jars were filled with 15% H_2_, O_2 _varying from 0-12.5%, and a balance of N_2 _to a total internal pressure of 1 atm. Incubation was carried out at 37°C for 72 hours. Plates were photographed in an inverted camera stage (Figure 5E). Colonies were identified and sized with an image analysis script implemented in MATLAB (MathWorks). Each data point represents size data collected from roughly 50 individual colonies.

### Hydrogenase mutagenesis and selection

Mutation libraries were created through error-prone PCR amplification of the hydrogenase genes with the GeneMorph II Random Mutagenesis Kit (Agilent Technologies). Exact primer sequences are indicated in additional file 1. The PCR products were digested with NotI and SpeI (for *ca*HydA) or NcoI and NotI (for *cs*HydA), purified, and ligated into a modified pCDFDuet-1 vector (Novagen). A target frequency of 4.5 mutations/kb was sought according to the manufacturer's instructions and confirmed by sequencing several clones plated nonselectively.

Host cells were transformed with the mutant hydrogenase libraries by electroporation and recovered in SOC media for 1 hour at 37°C. Following recovery, the cells were washed three times with Phosphate Buffered Saline (PBS) and plated on selective media. Approximately 10^7 ^unique transformants were applied to each plate, as assessed by serial dilution of the recovered cells. Selection plates were then transferred to Vacu-Quick jars prepared as above with a selective atmosphere containing 10% O_2 _and 15% H_2_. Incubation proceeded at 37°C for 4 days.

Selection restreaks were performed using Petri dishes with internal divisions to block the diffusion of metabolites from neighboring streaks[37]. Soft plastic inoculating loops were used to prevent marring of the agar surface and maintain a uniformly oxic environment[38,39].

### Structure and surface modeling

Homology models of hydrogenase mutants were constructed using SWISS-Model homology modeling service[40] using the *Clostridium pasteurianum *hydrogenase I structure[41] as a template (PDB ID: 3C8Y).

The protein surface was inferred with the NIH MBI Diffusion Accessibility Calculator[42]. The diffusion accessibility was averaged in a moving window of 5 residues, then thresholded to produce defined surface-accessible regions.

### Simulated mutation spectra

The set of point mutations isolated by our genetic selection was compared to the expected distribution under a null model of no selective pressure. The null distribution was generated as a parallel Monte Carlo simulation implemented in PYTHON. Our algorithm produced 5·10^9 ^randomly mutated sequences for each *ca*HydA and *cs*HydA in batches of 8 and 12, the number of variants of each enzyme isolated by the genetic selection. We used the point mutation spectrum provided by Agilent for the GeneMorph II kit and a total frequency of 4.5 mutations/kb. Nonsilent mutations were grouped by charge of the original and mutant amino acid (i.e. a positive to neutral substitution). Statistical 95% confidence intervals for the null distribution were derived from the size of each charge change group across simulated replicates.

## Results and Discussion

### Design for hydrogenase-dependent growth

**Figure 1:**
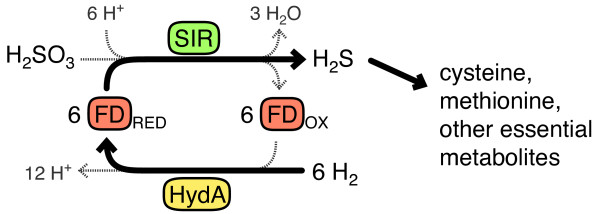
**A synthetic pathway linking hydrogenase activity to host viability**. A hydrogenase enzyme (HydA) consumes H_2 _and reduces ferredoxin (FD), a redox carrier protein. Ferredoxin then donates electrons to a plant-type sulfite reductase (SIR), which reduces sulfite to sulfide. With the native sulfite reductase deleted, this pathway becomes the only source of reduced sulfur, an essential metabolic precursor.

Figure 1 details the synthetic pathway designed for our selection and expressed in *E. coli*. An exogenous FeFe-hydrogenase consumes H_2 _and reduces ferredoxin. Ferredoxin donates electrons to sulfite reductase for the reduction of sulfite to sulfide. Sulfide serves in the host as an indispensible sulfur source for cysteine biosynthesis.

Ferredoxin, an electron-carrying iron-sulfur (Fe-S) protein, is the native redox partner of the best-characterized FeFe-hydrogenases[43]. A ferredoxin also receives electrons from photosystem I in plants, suggesting that it could be adapted to mediate light-driven H_2 _production. We chose ferredoxin as an intermediate with this future application in mind. A ferredoxin homolog, *fdx*, is found in *E. coli*, where it plays an essential role as a scaffold site for iron-sulfur cluster assembly[44]. *E. coli *does not appear to use ferredoxin as an electron carrier in the metabolic network, instead relying on NAD(P)H. We therefore hypothesized that ferredoxin chemistry would be insulated from native metabolism.

The KEGG pathway database[45] identifies three enzymes that produce essential metabolites using ferredoxin as an electron source. Glutamate synthase, nitrite reductase and sulfite reductase activities are all essential for the growth of *E. coli *in minimal medium. The native bacterial enzymes draw electrons from NADPH to produce glutamate, ammonia and sulfide, respectively. The analogous enzymes in plants yield the same products while drawing electrons from ferredoxin, a common redox carrier in those species. While all three products are indispensable for *E. coli *viability, sulfide is consumed in the smallest molar quantity[46]. We therefore chose to employ sulfite reductase, reasoning that the small metabolic requirement would afford more tolerance for suboptimal performance of the heterologous pathway.

Sulfite reductase is not essential on cysteine-containing rich media, but it becomes essential on selective media containing only oxidized sulfur sources such as sulfate or sulfite. In the absence of the native *E. coli *NADPH-dependent sulfite reductase, *cysI*, the synthetic pathway is the only metabolic source of reduced sulfur. If the components of this pathway are insulated from any endogenous electron sources, then hydrogenase activity will also be essential for growth. Increasing O_2 _concentrations, by inactivating the hydrogenase, will eventually inhibit the ability of host cells to grow on sulfite. This synthetic pathway therefore enables a genetic selection for hydrogenase mutants with an ability to support growth in high O_2_.

### Synthetic ferredoxin-dependent sulfite reduction

**Figure 2:**
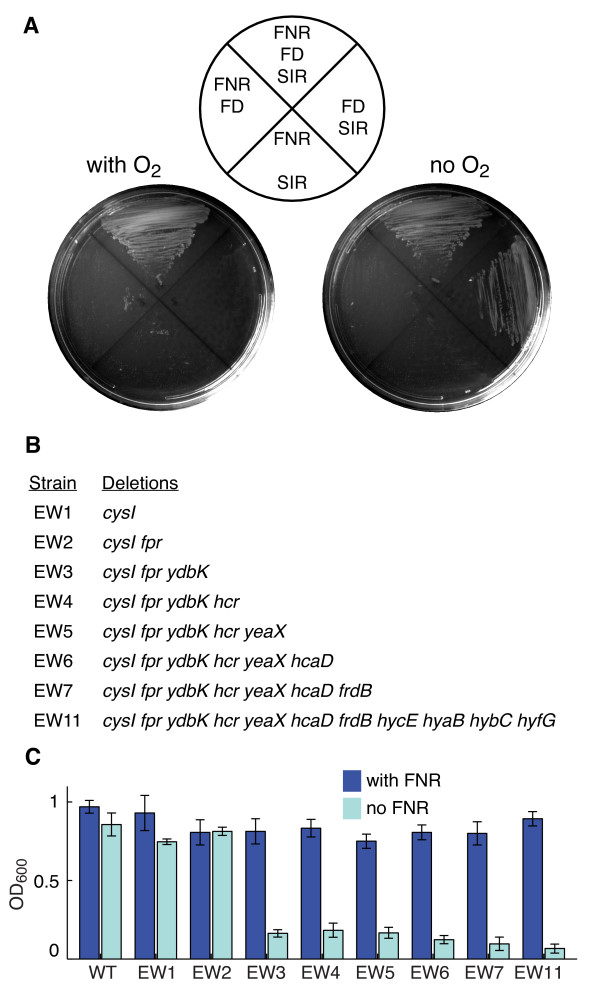
**Genetic insulation of the synthetic pathway**. A) *E. coli *BL21(DE3) *cysI *cells were transformed with plasmids expressing *zm*FNR, *so*FD and *zm*SIR. The *cysI *deletion conveys a requirement for reduced sulfur, which the heterologous pathway supplies. Cells were grown for 24 hours on selective media with or without atmospheric O_2_. All three factors were required to rescue growth under aerobic conditions (left plate). Expression of *zm*FNR was not required under anaerobic conditions (right plate), indicating that *so*FD was receiving electrons from another source. B) Genetic deletions were targeted to eliminate potential endogenous redox partners for ferredoxin, therefore linking sulfide production specifically to a synthetic electron source. Also deleted were the catalytic subunits of each native hydrogenase, ensuring that only exogenous H_2 _was present in our system. C) Each deletion strain was transformed with *so*FD, *zm*SIR and either *zm*FNR or an empty plasmid. Growth was measured after 18 hours at 37°C under strict anaerobic conditions, as described in the methods. Sequential deletions reduced the nonspecific anaerobic background growth, with the largest effect produced by the deletion of *ydbK*. The final deletion strain, EW11, showed no growth defect in rich media and was used in all later experiments.

We first sought to establish that the native *E. coli *BL21(DE3) sulfite reductase could be replaced with a ferredoxin-dependent pathway (Figure 2A). Deletion of the *cysI *sulfite reductase did not impair growth on rich media (LB), but eliminated growth on selective media with sulfate as the sole source of metabolizable sulfur. Expression of corn (*Zea mays*) sulfite reductase (*zm*SIR) alone, or together with spinach ferredoxin (*so*FD), failed to rescue growth. This indicated that the *E. coli *host provides no interacting source of reduced ferredoxin or ferredoxin-reductase activity under these conditions.

We then provided a heterologous source of reduced ferredoxin in the form of corn-derived (*Zea mays*) ferredoxin-NADP^+ ^reductase (*zm*FNR). This enzyme links *so*FD to the endogenous NADPH pool by catalyzing redox exchange between the two electron carriers [47]. FNR requires no maturation factors and is unaffected by O_2_, therefore it serves as a hydrogenase-independent control source of electrons for our pathway. Expression of *zm*FNR with *so*FD and *zm*SIR rescued growth of the *cysI *mutant on sulfate in aerobic selective media. The growth rescue required all three factors as well as IPTG induction of the expression plasmids. This result established that the ferredoxin and sulfite reductase components of our pathway were functional and insulated from native metabolism under aerobic conditions.

However, we found that heterologous *zm*FNR was not required to rescue growth when the selective strain was grown without ambient O_2 _(Figure 2A). While *so*FD and *zm*SIR expression were both still essential, this undesirable background growth indicated that the native metabolic machinery could donate electrons to ferredoxin in anoxic conditions. We did not observe anaerobic growth upon expression of *zm*SIR alone, indicating that electrons were entering the pathway through *so*FD.

Anaerobiosis effects global physiological adaptations in *E. coli*[48]. The transition from respiratory to fermentative growth is accompanied by a drop in cytosolic redox potential concomitant with an excess of reducing equivalents generated by glycolysis. A variety of metabolic pathways are expressed specifically in anaerobic conditions to dispose of electrons through electron acceptors including acetate, fumarate, nitrate and H_2_. Sulfite in our system represents a high potential electron sink, consistent with the increased tendency for electrons to enter the pathway under these conditions.

To produce the strictest possible connection between the heterologous pathway and strain fitness under selection, we sought to identify and eliminate any endogenous anaerobic electron sources. We selected 6 candidate interacting genes as potential nonspecific electron donors. Candidates were identified based on their homology to known ferredoxin-reducing proteins, with preference given to genes known to be induced anaerobically.

The following six candidate genes were deleted in the BL21(DE3) parent strain: *fpr*, *ydbK*, *hcr*, *yeaX*, *hcaD*, *frdB*. The genes were deleted serially, in a single host strain, in order to expose and eliminate potentially redundant or epistatic interactions of the candidate genes with our pathway (Figure 2B). The *fpr *gene encodes an anaerobic flavodoxin-NADP^+ ^reductase[49]. Overexpression of *ydbK*, a putative pyruvate:flavodoxin oxidoreductase, has been shown to drive hydrogenase activity through ferredoxin in *E. coli*[50]. The *hcr *locus encodes an anaerobically expressed NADH oxidoreductase that catalyzes the reduction of the hybrid cluster protein Hcp, an iron-sulfur protein with some homology to ferredoxin[51]. YeaX is a predicted oxidoreductase bearing Fe-S clusters that may associate with the ferredoxin-like YeaW. HcaD encodes a Ferredoxin:NAD^+ ^reductase involved in the degradation of 3-phenylpropionate. FrdB is an Fe-S protein involved in the anaerobic reduction of fumarate as a terminal electron acceptor. Within the 4-subunit menaquinol-fumarate oxidoreductase complex, FrdB shuttles electrons from the quinone pool to the catalytic flavoprotein FrdA[52].

We further deleted catalytic subunits of the three characterized endogenous hydrogenases, *hycE*, *hyaB *and *hybC*[53], and the putative but normally silent hydrogenase *hyfG*[54]. All native *E. coli *hydrogenases are of the NiFe class, unrelated to the FeFe class and therefore unlikely to interact directly with our pathway. However, eliminating all native hydrogenases ensures that any H_2 _production or consumption in our system could be attributed to the exogenous hydrogenase.

The knockout strains were transformed with *zm*SIR and *so*FD. Strains also received *zm*FNR as a synthetic electron source or an empty vector control. As described in the methods, growth was assayed anaerobically overnight in selective liquid media. The results of these experiments are shown in Figure 2.

The sequential knockout of candidate ferredoxin-interacting genes from the selection host improved the insulation of the test pathway from the endogenous redox pool. The first deletion, *fpr*, produced no measurable effect on strain growth. The largest contribution to the elimination of background growth came from the knockout of *ydbK*, with further deletions only modestly decreasing the background growth. These results are consistent with the effects of individual genes observed by Agapakis and colleagues[55]. The mutations had no individual or cumulative deleterious effect on strain growth when *zm*FNR was expressed. We designate as EW11 the final BL21(DE3)-derived strain, which bears the following complete genotype: *E. coli *B F^- ^*dcm ompT hsdS*(r_B_^- ^m_B_^-^) *gal *λ(DE3) *cysI fpr ydbK hcr yeaX hcaD frdB hycE hyaB hybC hyfG*. All subsequent experiments were performed in EW11 cells.

Sulfide production by our pathway was confirmed spectophotometrically by the formation of methylene blue, as described in the methods. Wild-type BL21 cells, cultured anaerobically in defined medium, produce small amounts of sulfide: 10 μM (± 10 at 95% confidence). No sulfide was detected in cultures of EW11 host cells, consistent with the deletion of *cysI*. Expression of *so*FD and *zm*SIR in EW11 resulted in sulfide accumulation only to a mean level of 2 μM (± 1). When zmFNR was also expressed as a source of electrons, sulfide levels increased dramatically to 200 μM (± 30). Similarly, when the *ca*HydA hydrogenase and maturation factors were expressed, sulfide levels reached 100 μM (± 27). Supplying the hydrogenase with atmospheric H_2 _further raised sulfide production to 160 μM (± 17). These results are consistent with the design of our pathway as a synthetic source of essential reduced sulfur.

### Biochemical hydrogenase O_2 _tolerance *in situ*

We sought to initiate our selection with a wild-type hydrogenase with the highest possible native activity and O_2 _tolerance. We reasoned this would improve the probability of evolving an enzyme with properties exceeding those described in nature. This also would allow us to perform our selection in the presence of some O_2_, reducing the observed anaerobic background growth. Biochemical techniques allow the *in vitro *determination of purified hydrogenase activity and O_2 _tolerance[20]. But because genetic selection can be performed only *in vivo*, we assayed hydrogenase O_2 _tolerance in cell lysates that approximate the cytosolic context.

*E. coli *expressing a hydrogenase derived from either *Clostridium acetobutylicum *(*ca*HydA), *Clostridium saccharobutylicum *(*cs*HydA), or *Chlamydomonas reinhardtii *(*cr*HydA), were grown to saturation in liquid culture under strict anaerobiosis. In each case the hydrogenase was coexpressed with the requisite maturation factors HydEF and HydG from *C. reinhardtii*, which are known to mature clostridial FeFe-hydrogenases[56]. Culture lysates were exposed to O_2 _for fixed periods of time and remaining hydrogenase activity was measured biochemically, as described in the methods.

**Figure 3:**
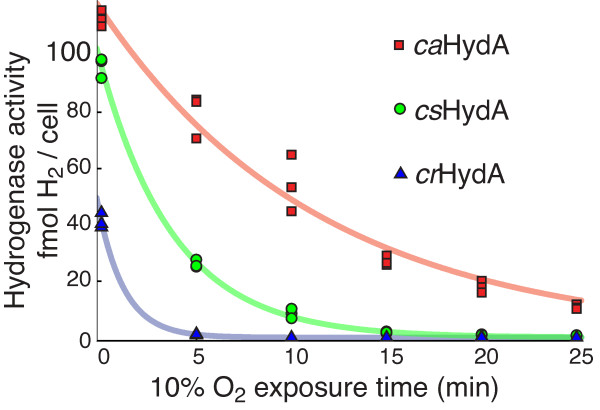
**O_2 _tolerance for three hydrogenases *in situ***. Cells expressing a hydrogenase from either *C. acetobutylicum *(*ca*HydA), *C. saccharobutylicum *(*cs*HydA) or *C. reinhardtii *(*cr*HydA) were exposed to 10% O_2 _at 1 atm total pressure for the indicated times. Remaining activity was assessed with methyl viologen, as described in the methods. Three biological replicates are plotted for each time point. Significant differences in both maximal activity and O_2 _tolerance were evident. Hydrogenase inactivation by O_2 _was well described by first order kinetics, and the best-fit exponential decay curves are shown. The *ca*HydA enzyme exhibited a characteristic half-life of 8 (± 0.8) minutes, the *cs*HydA enzyme 2.7 (± 0.2) minutes and *cr*HydA 1.0 (± 0.3) minutes, including 95% confidence intervals.

The three hydrogenases were found to differ in both O_2 _tolerance and maximal activity levels, as shown in Figure 3. The anaerobic activities of the clostridial hydrogenases, *ca*HydA and *cs*HydA, were comparable to each other and both substantially higher than the activity of the *Chlamydomonas *enzyme, *cr*HydA. While our assay controlled for cell density, the *in situ *context of our system did not account for possible differences in expression level, maturation or folding efficiency. Replacing the *C. reinhardtii *maturation factors with those derived from *C. acetobutylicum *yielded the same *in situ *activity for all three hydrogenases (not shown). While the *in situ *assay does not reflect biochemical specific activities, it measures the effective activity in *E. coli *expressing each hydrogenase, the relevant parameter for our genetic selection.

We also observed substantial variation in the natural O_2 _tolerance of the three enzymes. In each case the inactivation by O_2 _could be well-described by first-order reaction kinetics, resulting in an exponential decrease of activity with time. Exposure to 0.1 atm O_2 _partial pressure degraded activity of *ca*HydA with a characteristic half-life of 8 (± 0.8) minutes, including a 95% confidence interval. The *cs*HydA enzyme showed a half-life of 2.7 (± 0.2) minutes in O_2_, and *cr*HydA activity degraded still more rapidly, with a half-life of 1.0 (± 0.3) minutes. The half-life measurements are intensive biochemical properties of the enzymes *in situ*, independent of possible differences in hydrogenase expression or maturation levels.

The differences in O_2 _tolerance are striking. The clostridial enzymes *ca*HydA and *cs*HydA share 81% amino acid sequence similarity and nearly identical domain architecture. *C. acetobutylicum *and *C. saccharobutylicum *also inhabit similar strictly anaerobic ecological niches[57]. The algal *cr*HydA is more divergent, only 53% similar to *ca*HydA, yet shares the conserved catalytic domain. *Chlamydomonas*, a eukaryote, exhibits a generally aerobic metabolism. All three enzymes in this experiment receive identically assembled FeFe cluster active sites from shared maturation factors. Yet the half-life of *ca*HydA in O_2 _is twice that of *cs*HydA and 8 times that of *cr*HydA. Because *ca*HydA and *cs*HydA showed higher activity and superior O_2 _tolerance, we chose to focus on those enzymes for further study.

### The ferredoxin-hydrogenase interaction

**Figure 4:**
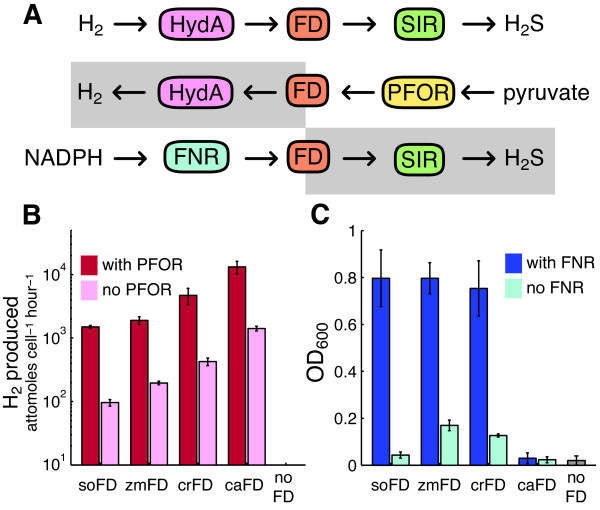
**Stepwise optimization of pathway design**. A) The proposed pathway operates in two steps, with electrons flowing first from HydA to FD, then from FD to SIR. Substituting PFOR for SIR allows an independent test of the HydA-FD interaction. Substituting FNR for HydA allows an independent test of the FD-SIR interaction. B) PFOR-driven H_2 _production *in vivo *with a panel of ferredoxins. H_2 _production in the presence of PFOR confirms the ability of a ferredoxin to couple with hydrogenase. H_2 _production in the absence of PFOR suggests that a ferredoxin is reduced by a nonspecific endogenous source, which would tend to confound our genetic selection. C) FNR-rescued growth with a panel of ferredoxins under anaerobic conditions. Growth with FNR confirms the ability of a ferredoxin to couple with SIR. FNR-independent growth suggests that a ferredoxin is not well insulated from endogenous redox sources. All experiments were conducted under anaerobic conditions with the EW11 strain, as described in the methods. Error bars are 95% confidence intervals.

The synthetic pathway we propose operates in two redox steps, hydrogenase to ferredoxin and ferredoxin to sulfite reductase. Each step must be both efficient and well-insulated for the overall design to be effective. We therefore devised independent *in situ *assays for each step of the pathway, as depicted in Figure 4A. By evaluating the performance of various ferredoxins in these assays, we sought to identify the ferredoxin best suited for a genetic selection. An optimal ferredoxin would demonstrate a robust interaction with both hydrogenase and sulfite reductase, while remaining insulated from nonspecific interactions with the endogenous redox pool.

We first tested the hydrogenase-ferredoxin connections, employing an independent source of electrons for ferredoxin. In this assay, adapted from Agapakis *et al*.[55], each ferredoxin is reduced by exogenous pyruvate-ferredoxin oxidoreductase derived from *Desulfovibrio africanus *(*da*PFOR), before transferring electrons to the hydrogenase. The overall activity of this pathway is measured as H_2 _production *in vivo*, described in the methods.

The direction of the hydrogenase reaction is the reverse of that sought for the final selection pathway, i.e. it is producing rather than consuming H_2_. FeFe-hydrogenases are known to operate reversibly, though they generally function to produce H_2 _in nature[58]. The production of H_2 _in this case is energetically driven by the highly favorable decarboxylation of pyruvate to acetyl-CoA[59]:

Whereas in the proposed selection pathway, the consumption of H_2 _is instead driven by the reduction of highly electronegative sulfite:

H_2 _production from *ca*HydA, driven by *da*PFOR and mediated by a panel of candidate ferredoxins is shown in Figure 4B. These data reflect both the ferredoxin-*ca*HydA interaction and the ferredoxin-*da*PFOR interaction. Because this assay does not account for variations in the ferredoxin-*da*PFOR interaction, it could not be used to infer quantitatively the efficiency of electron transfer between a ferredoxin and *ca*HydA. Each ferredoxin had the capacity to facilitate electron transfer to *ca*HydA, as shown by the substantial increase in H_2 _production relative to cells without exogenous ferredoxin. No H_2 _production was detectable without expression of a ferredoxin. Consistent with previously reported results[55], we observed by far the most hydrogenase activity with the native redox partner, *ca*FD.

Although expression of *da*PFOR significantly increased H_2 _production from each ferredoxin, it was not necessary for *in vivo *hydrogenase activity. H_2 _production in the absence of *da*PFOR is a measure of nonspecific electron flow from endogenous metabolism to a ferredoxin. Any such flow would tend to weaken the link between hydrogenase activity and host viability in a genetic selection. An optimal ferredoxin partner would produce high hydrogenase activity with *da*PFOR and no activity without *da*PFOR. We found nonspecifically driven H_2 _production to be significantly less than, yet proportional to, *da*PFOR-driven production for all ferredoxins.

### The ferredoxin-sulfite reductase interaction

Optimal function of the second step in our pathway requires a robust and specific redox exchange of the mediating ferredoxin with *zm*SIR. We therefore used *zm*FNR as an independent source of electrons to characterize the interaction between our panel of ferredoxins and the *zm*SIR. Growth was measured in anaerobic selective media for strain EW11 with and without *zm*FNR expression. The interactions with both *zm*FNR and *zm*SIR contribute to the ability of a given ferredoxin to facilitate growth in this system. As above, growth in the absence of *zm*FNR expression reflects the tendency of a ferredoxin to receive electrons nonspecifically from the endogenous redox pool.

The results of growth assays from the second test pathway are shown in Figure 4C. In contrast to the results of the H_2_-production assay, we found that the clostridial ferredoxin *ca*FD showed the worst performance in the sulfite reductase assay, producing no significant growth. Each of the other ferredoxins tested, *so*FD, *zm*FD and *cr*FD, were able to effectively rescue the sulfide auxotrophy. In the case of the *zm*FD and *cr*FD, we also observed significant background growth in the absence of *zm*FNR expression, indicating nonspecific interactions of these ferredoxins with native metabolism. The *so*FD produced no observable background growth under these conditions.

None of the ferredoxins tested performed optimally in both the sulfite reductase and hydrogenase interaction assays. In both cases the differences in performance may be attributed to the distinction between plant-type and bacterial-type ferredoxins. The *so*FD, *zm*FD and *cr*FD proteins all belong to the plant-type class of ferredoxins. These proteins carry electrons in a characteristic Fe_2_S_2 _active center[60]. In contrast, *ca*FD is a bacterial-type ferredoxin with a Fe_4_S_4 _cluster[61]. Plant and bacterial ferredoxins share structural and sequence homology[62,63] and can functionally substitute for one another in some cases[64]. However, it seems likely that the relative divergence of bacterial *ca*FD precludes an interaction with either *zm*FNR or *zm*SIR, both of which natively pair with plant-type ferredoxins.

We also noted an apparent tendency to higher background activities for ferredoxins with higher redox potentials. Reactions with electrons from the endogenous redox pool may become more thermodynamically favorable as the redox potential of the ferredoxin increases. Among the plant-type ferredoxins the *so*FD, with a redox potential of -420 mV[47], showed the lowest nonspecific activity in both assays. The *zm*FD at -345 mV[47] and *cr*FD at -390 mV[65], exhibited higher backgrounds. This is also consistent with the theory that ferredoxin interactions are governed by Fe-S cluster redox potentials[33,66].

We chose *so*FD as a best functional compromise for the design requirements of our pathway. This ferredoxin clearly demonstrates an ability to function in both redox steps, and showed the least nonspecific activity in each. It is also one of the biochemically best characterized ferredoxins and a model for ferredoxin-photosystem interactions[67].

### O_2 _sensitivity of hydrogenase-rescued *E. coli*

We next characterized *in situ *the behavior of the complete synthetic rescue pathway incorporating *so*FD, *zm*SIR and a hydrogenase electron source. In particular, we sought to quantify the effect of O_2 _on the growth of the selection strain in conditions as similar as possible to those that would be encountered in a genetic selection. Strain EW11 expressing *so*FD, *zm*SIR, and hydrogenase maturation factors was transformed with either *ca*HydA, *cs*HydA, or *cr*HydA. Cells were plated at low density on selective plates under custom atmospheres with varying O_2 _pressures. Growth was quantified by measuring the size of colonies formed after three days, as described in the methods.

**Figure 5:**
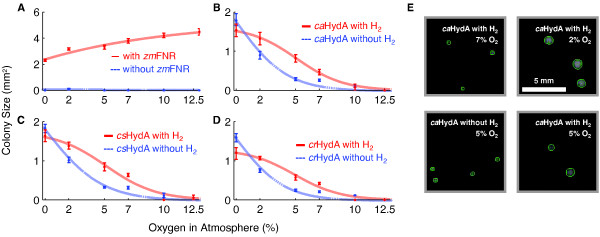
**Hydrogenase-rescued growth is O_2_-dependent and H_2_-dependent**. The genetically insulated EW11 strain expressing *so*FD, *zm*SIR, and hydrogenase maturation factors was transformed with *zm*FNR (A), empty vector (A), *ca*HydA (B), *cs*HydA (C), or *cr*HydA (D). Cells were plated at low density under custom atmospheres at varying O_2 _levels with and without H_2_. Colony sizes were measured after 3 days by imaging and automated analysis, as described in the methods. Representative images illustrating the effects of O_2 _and H_2 _on colony size are displayed in (E), with green circles indicating computationally identified colonies. Growth of strains rescued by each hydrogenase decreased monotonically with increasing O_2 _levels, becoming nearly undetectable at 10% O_2_. Atmospheric H_2 _improved growth but was not required for growth, suggesting an alternative source of reducing equivalents for the hydrogenase. Indicated for each curve is a best-fit sigmoid of the form area = a/(1+exp(b·(O_2_-c))). Error bars are 95% confidence intervals.

The dose-response relationship of O_2 _with various selection strains is depicted in Figure 5. Negative control strains expressing only *so*FD, *zm*SIR, and maturation factors showed no measurable growth under any atmosphere (Figure 5A). Positive control strains expressing the O_2_-tolerant *zm*FNR as an electron source showed robust growth under all conditions. Growth for the positive control tended to increase with increasing O_2_, consistent with the energetic advantages of aerobic metabolism in *E. coli*.

Hydrogenase-rescued strains showed O_2_-dependent growth (Figure 5B-D). For each hydrogenase, growth was the best in 0% O_2 _and decreased monotonically until almost no growth was detectable in 10% O_2_. Growth supported by a hydrogenase was always less than that observed with *zm*FNR, with colonies less than half as large forming even under strict anaerobiosis. The *ca*HydA enzyme was the most effective hydrogenase at supporting growth over all conditions, followed by *cs*HydA and *cr*HydA. This ordering is consistent with the *in situ *biochemical properties we determined previously. Interestingly, we detected only weak differences in the O_2_-dependent growth profiles of the hydrogenases relative to the differences in their *in vivo *activity levels and O_2 _tolerance. For example, *ca*HydA shows roughly two-fold higher activity and an eight-fold longer half-life than *cr*HydA. Yet the strain rescued with *cr*HydA produced colonies only about 30% smaller and reached 50% growth inhibition at the same O_2 _level.

Although growth in this assay was strictly dependent on hydrogenase expression, we found that growth did not require the addition of H_2 _to the atmosphere. Larger colonies were produced in the presence of H_2 _with all hydrogenases at most O_2 _levels, yet significant growth was still observed upon replacement of H_2 _with N_2_. Representative images illustrating the effect of H_2 _and O_2 _on colony size are shown in Figure 5E. H_2_-independent growth was more O_2_-sensitive, reaching 50% inhibition at roughly 2% ambient O_2 _for all hydrogenases, against 5% for H_2_-supported growth. When supplied with H_2_, strains expressing respectively *ca*HydA, *cs*HydA and *cr*HydA were half-maximally inhibited by O_2 _levels of 5.2% (± 0.5), 5.7% (± 0.4), and 6.1% (± 0.7), including 95% confidence intervals. Without H_2_, the same level of inhibition was respectively reached at O_2 _levels of 2.5% (± 0.1), 2.2% (± 0.2), and 2.3% (± 0.3). This growth could not be attributed to endogenous H_2 _production, as the EW11 strain lacks all native hydrogenase activity.

Ferredoxin choice strongly affected the growth of the selection host in the growth-response assay. The substitution of *zm*FD for *so*FD in this pathway resulted in much larger colony sizes by area (data not shown). Yet cells expressing *zm*FD also showed significant nonspecific background growth in the absence of *zm*FNR or *ca*HydA, consistent with results shown in Figure 4. We chose to pursue a genetic selection only in strains demonstrating strictly hydrogenase-dependent growth.

### Genetic selection isolates hydrogenases with modified surface properties

Variant libraries of the *ca*HydA and *cs*HydA enzymes were produced by error-prone PCR, as described in the methods. These libraries were used to transform EW11 cells expressing *so*FD, *zm*SIR and the hydrogenase maturation factors HydEF and HydG. Transformed cells were grown on selective media plates for 4 days under 10% O_2_, a non-permissive atmosphere for cells rescued by a wild-type hydrogenase.

Colonies that appeared on the selection plates after incubation were restreaked twice onto selective media. One restreak was regrown under a selective atmosphere at 37°C for 4 days, the second was grown on identical plates in open air (~21% O_2_). Colonies growing in open air were considered false positives, while colonies growing only in the selective atmosphere were considered candidates to host O_2_-tolerant hydrogenases. The hydrogenase expression plasmid was isolated from candidate colonies and retransformed into naïve selection hosts. Retransformed hosts were restreaked selectively as before to verify plasmid-linked, hydrogenase-dependent growth.

**Figure 6:**
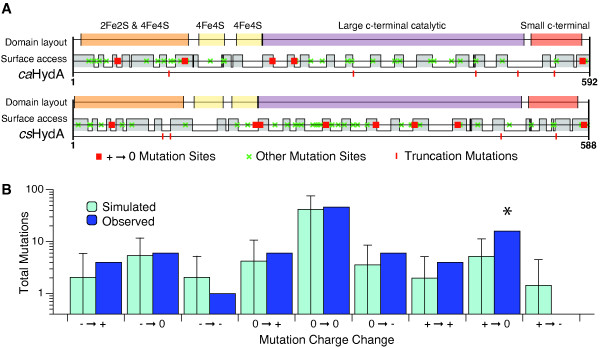
**Point mutations isolated by genetic selection**. A) Location of 110 nonsilent point mutations from 21 clones. Red squares indicate mutations that neutralize a positively charged residue, green xs mark mutations of all other classes, vertical lines indicate stop codons. Also indicated are the known hydrogenase structural domains and surface-accessible domains that were inferred as described in the methods. B) The observed and expected frequency of mutations by charge change class. Nonsilent mutations were grouped by the charges of both the original and mutant amino acid. Monte Carlo simulations, described in the methods, produce the expected frequency of each charge change class. As indicated by an asterisk, mutations in which a positively charged residue is replaced with a neutral are overrepresented (p-value: 1.7·10^-3^). Error bars are 95% confidence intervals.

Our genetic selection isolated twenty-three hydrogenases that passed all restreak and retransformation tests, containing a total of 110 nonsilent point mutations. These variants were sequenced and tested *in vitro *for hydrogenase activity as described in the methods. Of the twenty-three hydrogenases tested, only two retained full activity. A further nine hydrogenases retained some H_2_-evolution activity. The largest group of mutants, 12 of the 23, produced no detectable H_2_. The locations of the mutations to the *ca*HydA and *cs*HydA hydrogenase structures are shown in Figure 6. Full sequence and activity data for the mutants are provided in additional file 3.

Seven of the partially functional hydrogenases retained complete structures, containing only point mutations. However, two of the partially functional hydrogenases were truncated by stop codons introduced into their coding sequences. The *cs*HydA variant BB22 lacked 33 residues, removing part of the catalytic domain, yet possessed ≈ 5% of wild-type activity. The *ca*HydA mutant BB05 lacked 82 residues yet still produced detectable H_2_. To our knowledge, this is the first report that such a severely truncated hydrogenase, lacking the small C-terminal subunit, remains capable of H_2 _production activity and electron transfer.

Five of the H_2_-nonproducing hydrogenase mutants displayed only point mutations, while seven showed truncated catalytic domains. Interestingly, four of these seven mutants completely lacked the catalytic domain, containing only the N-terminal ferredoxin-like domains. The most truncated mutant that passed the selection, BB12, retains only 103 of 574 residues, and contains at most two of the four ferredoxin-like domains normally present.

A recurring motif was the modification of positive charges, as illustrated in Figure 6. Of the 110 nonsilent point mutations identified by the assay, 18 altered lysine residues. Of these 18, 4 were mutated to arginine: retaining the positive charge of the lysine residue, but substituting a larger side chain. An additional 5 lysine residues were mutated to asparagine, changing the charge to the residue to neutral. Residue 390 was mutated twice, once to isoleucine and once to asparagine, in both cases neutralizing the positive charge. Monte Carlo simulations, as described in the methods, confirmed that mutations neutralizing a positively charged residue were statistically enriched among our mutants (p-value: 1.7·10^-3^). Because each isolated variant contains multiple substitutions, we were not able to attribute functional changes to individual point mutations.

## Conclusions

The genetic selection for O_2 _tolerance produced no hydrogenases with O_2 _tolerance exceeding that of the wild-type, and only one with a comparable activity level. Given the observed widespread loss of H_2_-production activity, we speculate that the mutants identified by genetic selection confer growth by enhancing H_2_-independent electron transfer to ferredoxin. Consistent with this hypothesis are the results of Figure 5, indicating that hydrogenase, but not H_2_, is essential for growth under selection.

We found significant enrichment of mutations neutralizing positive surface charges of the hydrogenase. Electrostatic forces are known to have an important role in the kinetics and specificity of intermolecular interactions[68,69]. Ferredoxin proteins such as those used in our pathway display numerous and conserved negatively charged surface residues, which are thought to govern the specific recognition of various ferredoxin redox partners[66]. Site-directed mutagenesis of lysine residues on the surface of *Anabaena *FNR was found to block interaction with its native ferredoxin[70]. Our selection pathway employs a non-native pairing of spinach ferredoxin and clostridial hydrogenase, invoking suboptimally coadapted electrostatic interactions. The mutation of surface lysines may enhance ferredoxin-hydrogenase charge complementarity[71]. This could allow for more efficient electron transfer to ferredoxin, an essential activity for host viability.

Our selection also isolated a number of highly truncated hydrogenase variants which were found to retain some function. Variants BB22 and BB05 lacked portions of the small C-terminal hydrogenase subunit but were still competent for H_2 _production. Similarly truncated putative hydrogenases have been identified in the termite hindgut metagenome[72]. Even more severely truncated variants showed no hydrogenase activity, but were nevertheless capable of rescuing growth. Variant BB09, for example, retained only the N-terminal ferredoxin-like domains, which were nevertheless sufficient to facilitate electron transfer to our synthetic pathway. Future work to identify the minimal structural elements required for hydrogenase function may help to structurally integrate the enzymes with designed electron flows.

Our pathway allows O_2_-tolerant electron sources such as *zm*FNR to be distinguished from O_2_-sensitive sources such as *ca*HydA by their effects on host fitness. Therefore an O_2_-tolerant hydrogenase, once produced, could in principle be isolated using our selection. That no such hydrogenase was found suggests that other mutations exist to alter hydrogenase redox activity independently of the described O_2_-sensitive catalytic core[26]. Mutations of this sort may be more common than those specifically altering properties of the active site. The evolution of O_2 _tolerance may also require more simultaneous mutations than were sampled here, or more extensive structural alterations. The structural features that optimize O_2 _tolerance might also change when the direction of hydrogenase activity favors consumption versus production. Future efforts may benefit from combining genetic selection with high-throughput techniques to biochemically characterize hydrogenases, currently in development[73].

The data presented in Figure 5 suggest that each hydrogenase can be reduced by an unknown endogenous electron source other than H_2_. This electron source is eliminated by O_2_, but apparently through a different mechanism than that which directly inactivates the catalytic H-cluster[26]. Such a model would explain the apparent discrepancy between our *in vivo *and *in vitro *O_2 _tolerance assays. While Figure 2 shows different kinetics for the O_2 _inactivation of each hydrogenase *in vitro*, Figure 5 shows all three enzymes support comparable O_2_-tolerant growth. Hydrogenase activity in our system might also be limited by O_2 _sensitivity of the maturation factors, rather than the mature enzymes. To our knowledge, potential interactions of HydEFG with O_2 _have not yet been directly examined. An *in vivo *selection system would become even more valuable in such a case, as mutagenesis and selection could naturally be extended to the maturation factors.

We have shown that engineering can successfully insulate a synthetic electron transfer pathway from the endogenous *E. coli *redox pool. Minimizing losses to the cell through insulation of an artificial pathway allows more rational control of an engineered metabolic flux. We have demonstrated the use of convenient *in vivo *assays to validate isolated components of a synthetic H_2 _metabolism. The results of our assays revealed trade-offs in the choice of pathway components, allowing compromises to meet design goals. Finally, we have successfully tied an essential part of cellular metabolism, the synthesis of cysteine, to hydrogenase activity. By eliminating or disabling this activity with O_2_, we can halt cellular metabolism. These results demonstrate the utility of this pathway in a genetic selection for O_2_-tolerant hydrogenases. We anticipate that future work to characterize the H_2_-independent hydrogenase activity, and to optimize ferredoxin-hydrogenase electron transfer, will allow for more strict selection of H_2 _catalysts with desired properties.

## List of Abbreviations

*ca*: *Clostridium acetobutylicum*; *cr: Chlamydomonas reinhardtii*; *cs*: *Clostridium saccharobutylicum*; *da*: *Desulfovibrio africanus*; FNR: Ferredoxin-NADPH reductase; HydA: Hydrogenase; PFOR: Pyruvate-ferredoxin oxidoreductase; SIR: Sulfite reductase; *so*: *Spinacia oleracea*; *zm *- *Zea mays*.

## Competing interests

EHW, PMB, CMA, and PAS are inventors of International Patent Application No. PCT/US2009/058361, "In-Vivo Selection System For ID Iron (FEFE) Hydrogenase Properties."

## Authors' contributions

EHW designed experiments and drafted the manuscript. EHW, BB, CMA, PMB and GG performed experiments. EHW, BB, CMA, PMB, GG and PAS analyzed data. All authors read and approved the final manuscript.

## Supplementary Material

Additional file 1 **Vector sequences**. Contains complete sequences for all vectors used in this study and described in table 1. Click here for file

Additional file 2 **Media recipes**. Contains exact formulations of the selective and induction media used in these experiments. Click here for file

Additional file 3 **Mutant summaries**. Contains mutation, truncation and activity data for all mutants isolated in this work. Click here for file
