# Supplementary material for: A synthetic system links FeFe-hydrogenases to essential E. coli sulfur metabolism
Source: J Biol Eng. 2011 May 26;5:7. doi: 10.1186/1754-1611-5-7 (PMC3130634; doi:10.1186/1754-1611-5-7)
Supplement: Additional file 2 — Media recipes. Contains exact formulations of the selective and induction media used in these experiments. [file 1754-1611-5-7-S2.DOC]

# Additional file 2: Media recipes

## Selective Media

Selective media was a standard M9 formulation, supplemented with additional glucose, sulfate, ferric iron and a rich mix of amino acids less cysteine and methionine.

15 g Agar

12.8 g Na_2_HPO_4_⋅7H_2_O

3 g KH_2_PO_4_

0.5 g NaCl

1 g NH_4_Cl

0.1 mL 1M CaCl_2_

10 mL 1M MgSO_4_

25 mg Ferric citrate

0.5 g Sulfur dropout powder

100 mL 20% Glucose

1M IPTG 1mL

Sulfur dropout powder is a rich supplement mix with cysteine and methionine omitted:

| Amino acids | | | |  | Nucleotide bases | |
| --- | --- | --- | --- | --- | --- | --- |
| Alanine | 2.0 g | Leucine | 10.0 g |  | Adenine | 0.5 g |
| Arginine | 2.0 g | Lysine | 2.0 g |  | Uracil | 2.0 g |
| Asparagine | 2.0 g | Methionine | 0.0 g |  |  |  |
| Aspartic acid | 2.0 g | Phenylalanine | 2.0 g |  | Vitamins | |
| Cysteine | 0.0 g | Proline | 2.0 g |  | p-Aminobenzoic acid | 0.2 g |
| Glutamic acid | 2.0 g | Serine | 2.0 g |  | Inositol | 2.0 g |
| Glutamine | 2.0 g | Threonine | 2.0 g |  | Adenine | 0.5 g |
| Glycine | 2.0 g | Tryptophan | 2.0 g |  | Uracil | 2.0 g |
| Histidine | 2.0 g | Tyrosine | 2.0 g |  |  |  |
| Isoleucine | 2.0 g | Valine | 2.0 g |  |  |  |

## Induction media

Induction media for hydrogenase expression was LB with added glucose, ferric iron, phosphate buffer and Baker's antifoam reagent.

5 g Bacto tryptone

2.5 g Bacto yeast extract

5 g Sodium chloride

1.2 g KH_2_PO_4_

7.2 g K_2_HPO_4_

2.5 mL 20% Glucose

12.5 mg Ferric citrate

1 mL 1M IPTG

50 μL Baker’s antifoam B
